# The Value of Disinfectants

**Published:** 1921-12

**Authors:** 


*To the Editor of* THE HOSPITAL AND HEALTH REVIEW.

### The Value of Disinfectants.

*To the Editor of* THE HOSPITAL AND HEALTH REVIEW.

SIR,—I read a letter in your November number from the Sanitas Company, in which they say that between five and eight million gallons of disinfectant fluid were used by the Allied armies during the war, "with the result that the armies were kept practically free from infectious disease." So it would seem that disinfectants were one of those things that won the war. The war was won by tanks, by aeroplanes, by gas, by the rum ration—even by the cavalry, and, as a humorist has suggested, by the staff! But apparently it was five to eight million gallons of disinfectant that really did it. Surely, however, it is strategically unsound to publish important military secrets such as this. In the meantime, it may interest your correspondent to know that I kept a battalion quite healthy for many a long month without the use of any fluid disinfectants; and I think it is much better to keep things clean than to splash disinfectants about to cover dirt and to drown unpleasant smells.

CAPTAIN R.A.M.C.
(Temporary Commission.)

